# MR arthrographic analysis of labral and tendinous lesions in patients with shoulder avulsion injury

**DOI:** 10.1186/s12891-025-09217-3

**Published:** 2025-10-08

**Authors:** Yusuf Yahsi, Hayri Ogul, Rodi Ertogrul, Zakir Sakci, Yusuf Sulek, Mecit Kantarci

**Affiliations:** 1 Department of Orthopedic Surgery, Medical Faculty, Istanbul Medipol University, Istanbul, Turkey; 2 Department of Radiology, Medical Faculty, Istanbul Medipol University, Istanbul, Turkey; 3 Department of Radiology, Umraniye Training and Research Hospital, Health Sciences University, Istanbul, Turkey; 4 Department of Orthopedic Surgery, Sisli Hamidiye Etfal Training and Research Hospital, Health Sciences University, Istanbul, Turkey; 5 Department of Radiology, Medical Faculty, Ataturk University, Erzurum, Turkey

**Keywords:** Shoulder, Avulsion, Fracture, SLAP lesion, MR, Arthrography

## Abstract

**Objectives:**

This study aimed to evaluate the frequency and characteristics of specific labral lesions (SLAP and Bankart) and associated tendinous injuries in patients with avulsion fractures of the shoulder using magnetic resonance (MR) arthrography and to investigate their potential clinical implications.

**Methods:**

A retrospective review was performed on 850 shoulder MR arthrograms retrieved from our institutional PACS database.The study comprised a total of 35 patients with confirmed avulsion fractures and 30 age- and sex-matched controls. . All patients received both conventional MR imaging and 3D volumetric MR arthrography sequences. The evaluation focused on labral lesions (SLAP, Bankart), rotator cuff injuries and biceps tendon pathology which were systematically evaluated by two experienced radiologists.

**Results:**

The avulsion fracture of the greater tuberosity was the most commonly observed, accounting for 60% of cases. Labral pathology was considerably more prevalent among the patient cohort (51.4%) compared to the control group (23.3%) (p=0.039). Superior labrum anterior to posterior (SLAP) lesions were the most frequently identified labral abnormalities, and were significantly more common than both Bankart lesions (17.1%) and Bankart variant lesions (2.9%) (p=0.015). Patients presenting with fractures of the lesser tuberosity demonstrated a notably higher incidence of combined rotator cuff and biceps tendon pathology (77.8%) in contrast to those with greater tuberosity fractures (14.3%) (p=0.002).

**Conclusions:**

The research underscores a robust link between avulsion fractures and superior labral injuries, especially SLAP lesions. Moreover, there was a significant correlation between lesser tuberosity fractures and extensive tendinous pathology, indicating a greater degree of biomechanical compromise. These results highlight the critical role of MR arthrography in identifying concurrent labral and tendinous injuries that could affect clinical decision-making.

## Introduction

The shoulder joint, renowned for its intricate and complex anatomical structure, is notably susceptible to a wide variety of both soft tissue and bony injuries that may arise as a result of traumatic events[1, 2]. Avulsion fractures occurring in the shoulder region, especially those impacting the glenoid rim or the greater tuberosity, can significantly impair the joint's stability and overall functionality[3]. Moreover, labral and tendinous lesions often accompany these particular types of fractures, further exacerbating issues related to persistent joint instability and contributing to chronic pain[4].

The labroligamentous complex is integral to the stabilization of the shoulder joint and is a key factor in the development of shoulder instability[5]. The labrum constitutes a fibrocartilaginous ring surrounding the glenoid cavity, whereas the glenohumeral ligaments play a crucial role in ensuring both dynamic and static stability[6]. Damage to these components can result in recurrent shoulder dislocations and cause functional deficits. While conventional radiographic imaging is frequently sufficient for assessing bone structures, it falls short in evaluating soft tissue injuries and the condition of labroligamentous structures. Non-arthrographic magnetic resonance (MR) imaging of the shoulder joint can be used to observe many anatomical structures, such as the subarticular bone marrow, muscular structures, biceps tendon, and rotator cuff tendons. However, MR arthrography is mostly used for demonstrating labral pathologies complex abnormalities, and it is the most accurate imaging method established[7–9]. Moreover, a three-dimensional (3D) T1 weighted volumetric interpolated breath-hold examination (VIBE) MR arthrography with fat suppression allows multiplanar reconstruction using thinner image slices and provides good contrast for the surrounding soft tissues and bone structures[7, 10, 11].

 While previous studies have predominantly concentrated on osseous morphology, few have systematically evaluated concurrent labral and tendon lesions associated with shoulder avulsion fractures using MR arthrography. This research seeks to assess the labroligamentous structure damages associated with avulsion fractures of the shoulder joint utilizing comprehensive MR arthrography. The anticipated outcomes aim to advance the comprehension of shoulder instability and inform clinical practice by enhancing diagnostic and therapeutic methodologies.

## Material and methods

This investigation conducted a retrospective analysis of MR arthrograms from 850 patients who attended our clinic for shoulder MR arthrography between December 2010 and February 2025. The MR arthrograms were sourced from the hospital's Picture Archiving and Communication System (PACS) and meticulously assessed for the presence of avulsion fractures.

**Fig. 1:**
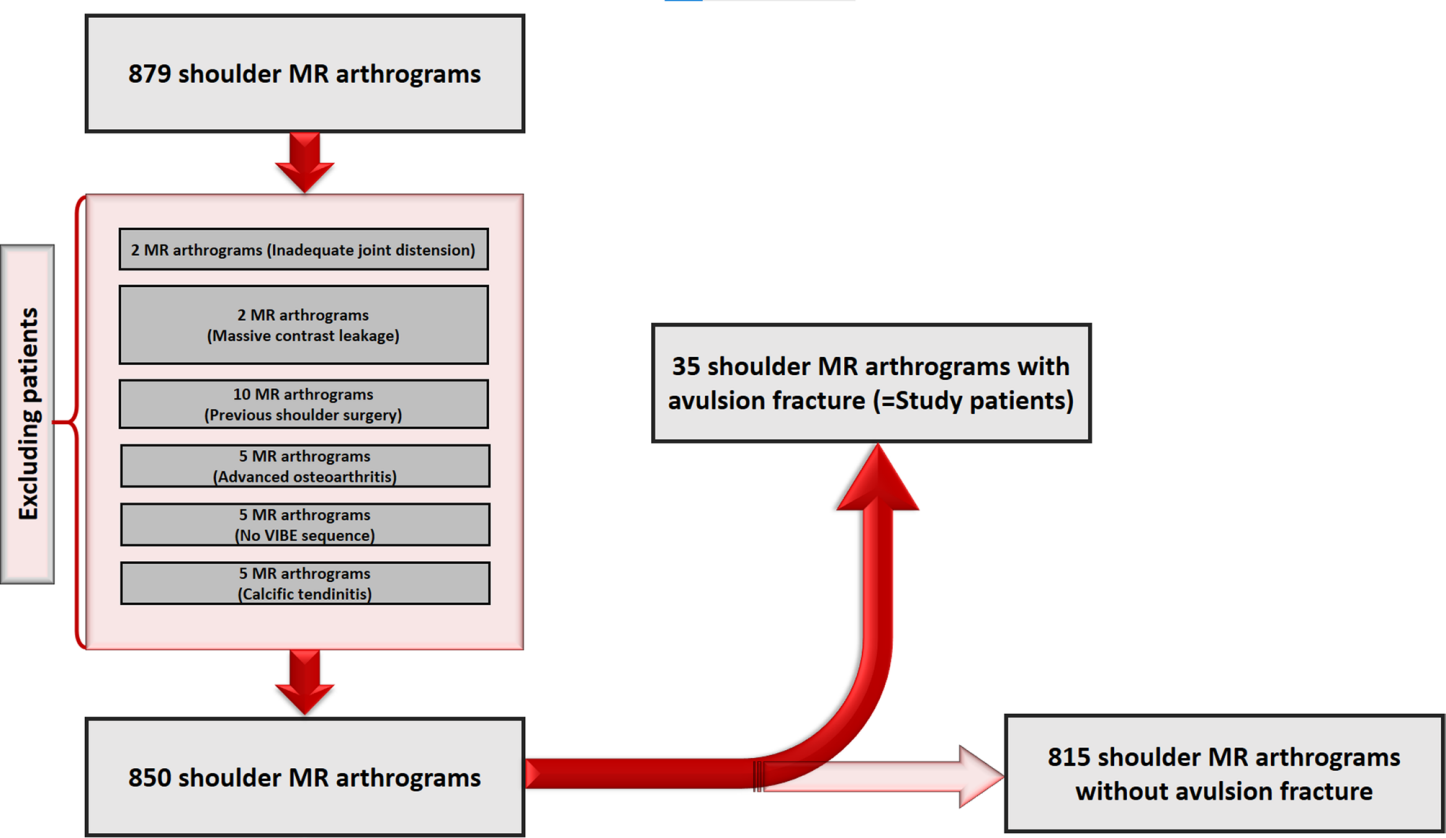
A flow diagram illustrating exclusion criteria

Participants qualified for inclusion in the study if they presented with either displaced or non-displaced avulsion fractures at tendon insertion sites of the greater and lesser tuberosities of the humerus, the coracoid process, the acromion, or the clavicle. Exclusion parameters included inadequate joint distension, significant leakage of contrast material into the pericapsular area, a history of previous shoulder surgery, and the presence of advanced osteoarthritis. Since the VIBE MR arthrography sequence is optimal for evaluating osseous structures near the shoulder joint, all avulsion fractures were determined exclusively using this method[12]. Patients lacking VIBE MR arthrograms were excluded. Moreover, individuals exhibiting MR and radiographic evidence indicative of calcific tendinitis were omitted from consideration. A flow diagram illustrating exclusion criteria is shown in figure 1. The study protocol received approval from the institutional ethics review board (Istanbul Medipol University/Non-Interventional Clinical Research Ethics Committee), and all participants provided written informed consent for the arthrography procedure.

 All MR arthrograms that met the inclusion criteria were retrospectively evaluated by a senior radiologist with 18 years of expertise in musculoskeletal imaging to ascertain the presence or absence of avulsion fractures. When an avulsion fracture was detected, its anatomical site was documented. Following this, patients with confirmed avulsion fractures on VIBE MR arthrography underwent further evaluation for concomitant pathologies by two radiologists with 18 and 6 years of experience in arthrographic imaging. The identification of additional pathologies was determined through a consensus between the two radiologists. To reduce potential biases, the control group was matched for both age and sex, and individuals with an initial diagnosis of instability were expressly excluded. The statistical analysis aimed to ascertain whether the presence and classification of avulsion fractures showed significant variations based on age, sex, and laterality (right vs. left shoulder). Furthermore, the prevalence of concomitant pathologies was compared between the patient and control cohorts.

 Statistical analyses were conducted using IBM SPSS Statistics version 22, based in Turkey. The Shapiro-Wilk test was employed to evaluate the normality of the data distribution. To describe the dataset, descriptive statistics such as mean, standard deviation, and frequency distributions were utilized. For comparative analysis of variables exhibiting normal distribution between groups, a one-way analysis of variance (ANOVA) was performed, followed by Tamhane's T2 post hoc test for pinpointing specific group differences. The Student’s t-test was used for comparing normally distributed variables in pairs. To analyze categorical data, Fisher’s Exact test, the Fisher-Freeman-Halton test, and the Continuity Correction (Yates) test were applied. A p-value of less than 0.05 was regarded as indicative of statistical significance.

## Results

In this investigation, MR arthrograms were assessed for 35 patients in the case group and 30 individuals in the control group. Within the patient group, 28 participants (80%) were male and 7 (20%) were female, whereas in the control group, 23 participants (76.7%) were male and 7 (23.3%) were female. In the patient cohort, MR arthrography was conducted on the right shoulder for 17 patients (48.6%) and on the left shoulder for 18 patients (51.4%). In the control group, MR arthrography was performed on the right shoulder for 18 individuals (60%) and on the left shoulder for 12 individuals (40%). All subjects underwent both conventional MR and MR arthrography sequences, along with three-dimensional (3D) VIBE MR arthrography. Additionally, CT arthrography was performed on 8 patients in the case group.

**Fig. 2:**
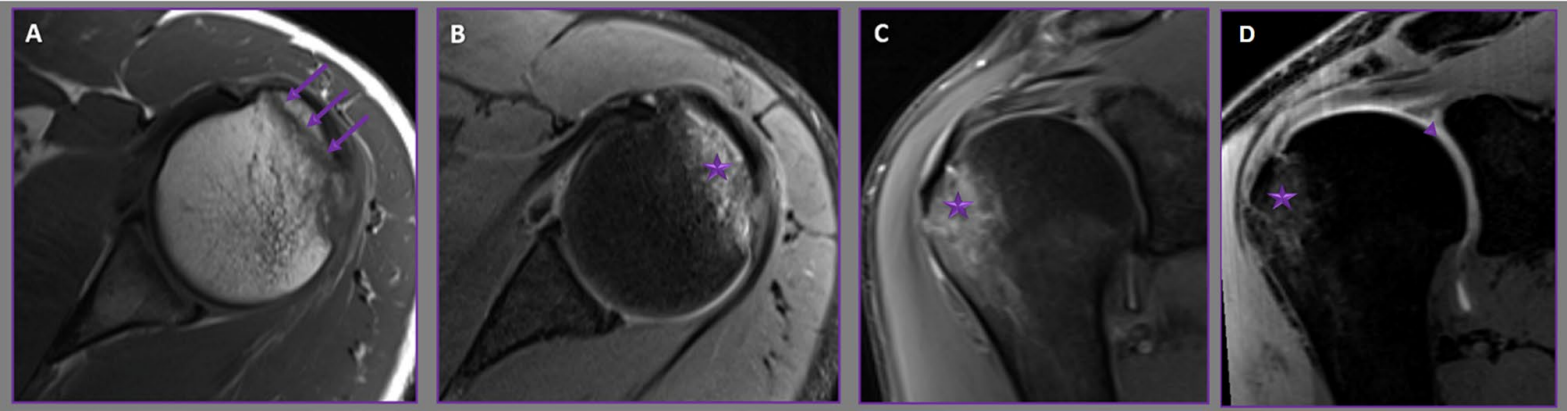
Axial T1 **(A)**- and PD-weighted**(B)** and oblique coronal PD-weighted **(C)** MR images demonstrate greater tuberosity avulsion fracture (arrows) and its associated bone marrow edema (stars). Oblique coronal fat-suppressed T1-weighted VIBE MR arthrogram **(D)** shows greater tuberosity avulsion fracture (star) and its associated SLAP lesion (arrow head)

**Fig. 3:**
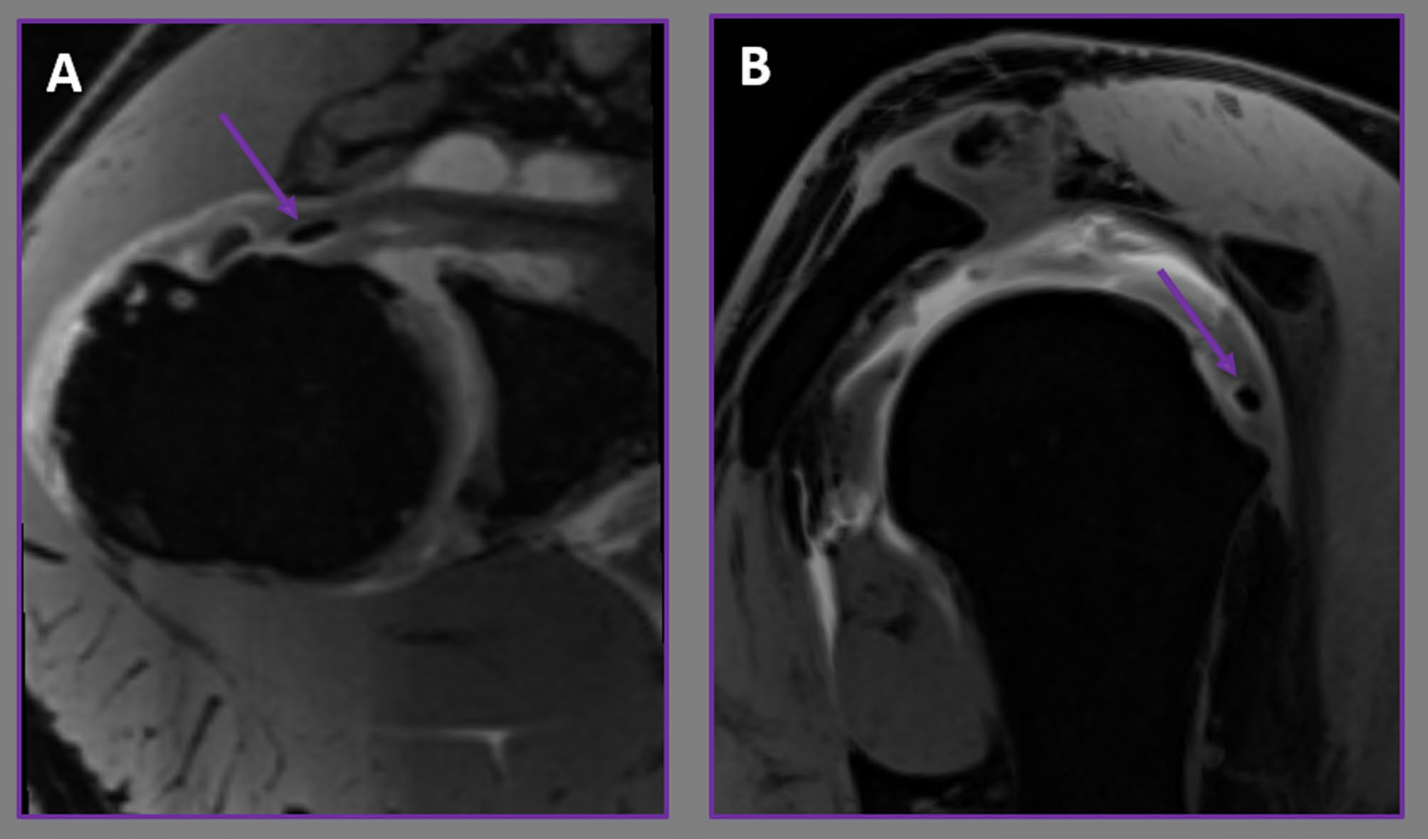
Axial **(A)** and oblique sagittal **(B)** fat-suppressed T1-weighted VIBE MR arthrograms show lesser tuberosity avulsion fracture (arrows) and its associated rotator cuff tendon rupture

 Within the group of 35 patients, the most commonly identified avulsion fracture was that of the greater tuberosity (Figure 2), occurring in 21 individuals (60%). Among those presenting with lesser tuberosity fractures, 77.8% also had associated rotator cuff tendon tears and biceps tendon issues (Figure 3). This association was statistically significantly greater compared to patients with greater tuberosity fractures (p = 0.002; p < 0.05). Table 1 provides a summary of the correlation between avulsion fracture types and tendon tears.

**Table 1:** Evaluating association between fracture types and rotator cuff tendon tear types

		Fracture type				*p*
		Great tuberosit	Minor tuberosit	Coracoid process	Acromion	
		n (%)	n (%)	n (%)	n (%)	
Associated tendon pathology	None	5 (%23,8)	0 (%0)	3 (%100)	0 (%0)	^1^0,002*
	Supraspinatus	9 (%42,9)	0 (%0)	0 (%0)	0 (%0)	
	Infraspinatus	1 (%4,8)	0 (%0)	0 (%0)	0 (%0)	
	Multiple rotator cuff	3 (%14,3)	2 (%22,2)	0 (%0)	0 (%0)	
	Rotator cuff + biceps	3 (%14,3)	7 (%77,8)	0 (%0)	2 (%100)	

^1^Fisher Freeman Halton Test

*p < 0.05

 A statistically significant variation in the average ages across different fracture types was identified (p = 0.010; p < 0.05) (Table 2). Subsequent post hoc pairwise analyses indicated that individuals without fractures exhibited a notably lower mean age compared to those with lesser tuberosity and acromion fractures (p₁= 0.037; p₂= 0.000; p < 0.05). Furthermore, the mean age of individuals with greater tuberosity fractures was significantly less than that of individuals with lesser tuberosity and acromion fractures (p₁= 0.003; p₂= 0.000; p < 0.05). No statistically significant age differences were detected among other fracture categories (p >0.05). Additionally, post-hoc power analysis demonstrated 69% statistical power for any labral pathology (absolute difference Δ = 28.1%, 95% confidence interval [CI] = –5.4% to 55.2%, Cohen’s h = 0.59), 78% statistical power for superior labrum anterior to posterior (SLAP) lesions (Δ = 24.8%, 95% CI = –2.8% to 46.1%, Cohen’s h = 0.67), and 77% statistical power for Bankart lesions (Δ = 17.1%, 95% CI =–3.2% to 32.7%, Cohen’s h = 0.85).

**Table 2:** Evaluating association between age, gender, and side and fracture types

		Fracture type					*p*^1^
		None	Great tuberosit	Minor tuberosit	Coracoid process	Acromion	
		(Min-Max)-(Mean ± SD)	(Min-Max)-(Mean ± SD)	(Min-Max)-(Mean ± SD)	(Min-Max)-(Mean ± SD)	(Min-Max)-(Mean ± SD)	
Age		(21–76)-(47,73 ± 14,22)	(19–65)-(43,24 ± 12,95)	(50–76)-(59,78 ± 8,11)	(27–73)-(55,67 ± 25,01)	(67–70)-(68,5 ± 2,12)	0,010*
		n (%)	n (%)	n (%)	n (%)	n (%)	
Patient Gender	Male	23 (%45,1)	16 (%31,4)	7 (%13,7)	3 (%5,9)	2 (%3,9)	
	Female	7 (%50)	5 (%35,7)	2 (%14,3)	0 (%0)	0 (%0)	
	p^2^	1,000					
Shoulder side	Right	18 (%51,4)	7 (%20)	9 (%25,7)	0 (%0)	1 (%2,9)	
	Left	12 (%40)	14 (%46,7)	0 (%0)	3 (%10)	1 (%3,3)	
	p^2^	0,001*					

^1^One-Way anova Test

^2^Fisher Freeman Halton Test

*p<0.05

 No statistically significant variations were observed in the distribution of fracture types between male and female patients (p >0.05). Nonetheless, the incidence of greater tuberosity fractures was markedly lower in those with right shoulder involvement (20%) compared to individuals with left shoulder involvement (46.7%) (p = 0.001; p < 0.05).

**Fig. 4:**
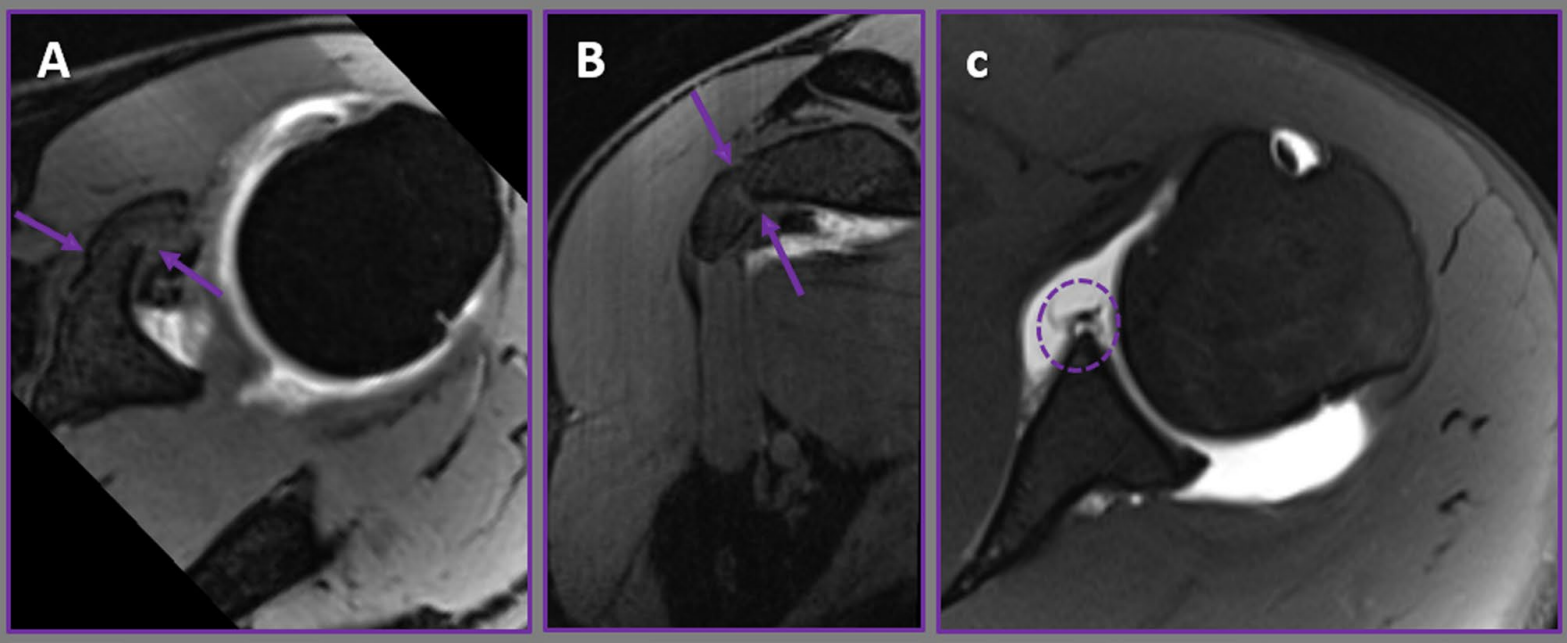
Axial **(A)** and oblique coronal**(B)** fat-suppressed T1-weighted VIBE MR arthrograms demonstrate an avulsion fracture (arrows) of the coracoid process. Axial T1-weighted SE **(C)** MR arthrogram shows associated Bankart lesion (circle)

 In the case cohort, labral pathology was identified in 18 (51.4%) MR arthrograms (Figure 4), compared to only 7 (23.3%) in the control cohort. This indicates a statistically significant higher prevalence of labral pathology in the patient cohort relative to the control cohort (p = 0.039; p < 0.05). Within the patient cohort, the most prevalent labral pathology was a superior labrum anterior and posterior (SLAP) lesion, detected in 11 cases (31.4%), which occurred with significantly greater frequency than in the control cohort (p = 0.015; p < 0.05). Comparative data regarding joint pathologies in the case and control cohorts are outlined in Table 3.

**Table 3:** Evaluating of the study parameters between both group

		Patient group	Control group	*p*
		(Min-Max)-(Mean ± SD)	(Min-Max)-(Mean ± SD)	
		n (%)	n (%)	
Patient Gender	Male	28 (%80)	23 (%76,7)	^2^0,981
	Female	7 (%20)	7 (%23,3)	
Shoulder side	Right	17 (%48,6)	18 (%60)	^2^0,502
	Left	18 (%51,4)	12 (%40)	
Fracture type	None	0 (%0)	30 (%100)	-
	Greater tuberosity	21 (%60)	0 (%0)	
	Minor tuberosity	9 (%25,7)	0 (%0)	
	Coracoid process	3 (%8,6)	0 (%0)	
	Acromion	2 (%5,7)	0 (%0)	
Associated tendon pathology	None	8 (%22,9)	9 (%30)	^3^0,707
	Supraspinatus	9 (%25,7)	10 (%33,3)	
	Infraspinatus	1 (%2,9)	1 (%3,3)	
	Biceps tendon long head	0 (%0)	1 (%3,3)	
	Multiple rotator cuff	5 (%14,3)	2 (%6,7)	
	Roator cuff + biceps	12 (%34,3)	7 (%23,3)	
Labral lesion	None	17 (%48,6)	23 (%76,7)	^2^0,039*
	Present	18 (%51,4)	7 (%23,3)	
Associated labrum pathology	None	17 (%48,6)	23 (%76,7)	^3^0,015*
	SLAP	11 (%31,4)	5 (%16,7)	
	Bankart	6 (%17,1)	0 (%0)	
	Bankart Varıant	1 (%2,9)	1 (%3,3)	
	Revers Bankart	0 (%0)	1 (%3,3)	
Glenohumeral ligament tear	None	9 (%25,7)	15 (%50)	^2^0,078
	Present	26 (%74,3)	15 (%50)	
Associated glenohumeral ligament pathology	None	9 (%25,7)	15 (%50)	^3^0,054
	SGHL	10 (%28,6)	9 (%30)	
	MGHL	0 (%0)	1 (%3,3)	
	IGHL	1 (%2,9)	1 (%3,3)	
	SGHL and MGHL	14 (%40)	4 (%13,3)	
	All	1 (%2,9)	0 (%0)	

^1^Student t Test

^2^Continuity (Yates) Düzeltmesi

^3^Fisher Freeman Halton Test

*p<0.05

## Discussion

The present study primarily concentrated on the evaluation of distinct labral pathologies, with a particular focus on SLAP and Bankart lesions, in addition to concomitant rotator cuff and biceps tendon injuries, in patients with incidentally diagnosed avulsion fractures who were undergoing MR arthrography. The outcomes revealed that the frequency of labral pathology was significantly higher in the patient group (51.4%) compared to the control group (23.3%) (p˂0.05; p=0.039). Notably, SLAP lesions were identified more frequently (31.4%) than Bankart lesions (17.1%) and Bankart variant lesions (2.9%) (p˂0.05; p=0.015), highlighting a strong association between avulsion fractures and superior labral injuries. These results are consistent with current literature, which also documents a high occurrence of labral tears in instances of traumatic shoulder injuries[14].

 In our study, a critical observation was the comparatively higher occurrence of SLAP lesions relative to Bankart lesions among patients with avulsion fractures. As reported by Barber et al., SLAP lesions generally result from continuous traction and compressive forces affecting the superior labrum.[13]The anatomical linkage between the superior labrum and the long head of the biceps tendon makes the superior labrum particularly susceptible to high-energy trauma such as avulsion fractures[15]. Consistent findings were reported by Brockmeyer et al. who found a higher prevalence of SLAP lesions compared to Bankart lesions in instances of traumatic shoulder injuries. This pattern may be attributed to the significant tensile forces exerted by the biceps tendon on the superior labrum during avulsion incidents.[16] Furthermore, SLAP lesions are frequently identified as a prevalent concern in young, physically active individuals, highlighting their importance in cases of traumatic shoulder instability[17]. These findings emphasize the need for a thorough examination of the superior labrum in patients with avulsion fractures, as undetected SLAP lesions could lead to persistent shoulder dysfunction and instability.

The diminished frequency of Bankart lesions and their variants observed in our study may be attributed to the injury mechanism characteristic of avulsion fractures. Although Bankart lesions are predominantly associated with anterior shoulder dislocations, superior labral anterior-posterior (SLAP) lesions have been documented in both dislocations and high-energy trauma events[18]. The significant association between avulsion fractures and SLAP lesions suggests that the superior labral structures may be particularly vulnerable to damage under these circumstances. This finding underscores the importance of comprehensive MR arthrography evaluations, focusing on the integrity of the superior labrum, for patients presenting with avulsion fractures.

Additionally, our research identified a significantly lower incidence of combined rotator cuff and biceps tendon pathology in patients with greater tuberosity fractures (14.3%) compared to those with lesser tuberosity fractures (77.8%) (p˂0.05; p=0.002). This implies that fractures of the lesser tuberosity may be correlated with more severe soft tissue injuries, aligning with findings from existing literature[11]. In elderly patients, rotator cuff tears are well-documented contributors to shoulder dysfunction and pain[19]. The substantial occurrence of rotator cuff pathology alongside lesser tuberosity fractures suggests these injuries may arise due to heightened stress on tendinous structures, underscoring their clinical significance.

We identified a statistically significant variation in mean age across different fracture types (p˂0.05; p=0.010), with fractures of the lesser tuberosity and acromion being more common among older individuals. This observation is consistent with existing research highlighting age-related alterations in cartilage thickness and bone density, which may make elderly individuals more susceptible to these fractures[20]. The degenerative changes, in conjunction with diminished bone mineral density, may elevate the risk of such fractures in the older population[21].

Furthermore, our results demonstrated a significantly lower incidence of greater tuberosity fractures in patients with right shoulder involvement (20%) as opposed to left shoulder involvement (46.7%) (p˂0.05; p=0.001). This disparity may stem from biomechanical variances between dominant and non-dominant shoulders. Existing literature posits that the dominant shoulder possesses superior neuromuscular control and mechanical robustness, rendering it less prone to traumatic fractures[22]. Our findings corroborate this theory, indicating that enhanced muscle stabilization in the dominant shoulder may offer increased protection against avulsion fractures.

Our study has several limitations. First, due to the retrospective nature of this study, our MR arthrography protocol was not specifically optimized for evaluating subtle labral and tendinousstructures that could contribute to shoulder instability. Second, most patient imaging was performed in a neutral shoulder position, which may limit the ability to assess certain dynamic instabilities. Positional MR arthrography could provide a more detailed evaluation of labral and ligamentous pathologies by demonstrating their behavior under stress. Third, joint distension variability may have influenced the visualization of intra-articular structures, potentially masking or exaggerating certain findings. Optimized joint distension techniques in future studies could enhance diagnostic sensitivity. Furthermore, the inclusion of elderly patients may represent a potential source of bias, as superior labral lesions are more prevalent in this demographic even in the absence of trauma. This factor must be considered when interpreting the high frequency of SLAP lesions in the present cohort. Lastly, our findings were not confirmed through arthroscopic or cadaveric studies, which are considered gold-standard methods for assessing intra-articular shoulder pathologies. Future prospective studies incorporating direct arthroscopic correlation would improve the accuracy and clinical applicability of our results. Although thin-section CT scanning is considered the gold standard imaging method for evaluating bone fractures, fat-suppressed 3D VIBE MR arthrographic imaging has been reported to have nearly equal results to non-arthrographic multislice CT in evaluating glenoid bone fractures[11]. Because our only 8 patients have CT arthrography images, we used fat-suppressed 3D VIBE MR arthrography for evaluating shoulder avulsion fractures.

In summary, this study offers significant insights into the correlation between avulsion fractures and associated labral and tendinous pathologies. The notably higher prevalence of SLAP lesions relative to Bankart lesions emphasizes the necessity of superior labral assessment in such injuries. Furthermore, the connection between lesser tuberosity fractures and severe soft tissue pathology underscores the importance of thorough imaging evaluations in these patients. Future research with larger sample sizes is recommended to delve deeper into the biomechanical and clinical ramifications of these findings and to refine treatment strategies for individuals with avulsion fractures.

## Data Availability

The data that support the findings of this study are not openly available due to reasons of sensitivity of human data and are available from the corresponding author upon reasonable request.

## Abbreviations

CT: Computed tomography
MR: Magnetic resonance
SLAP: Superior labrum from anterior to posterior
VIBE: Volumetric interpolated breath-hold examination
